# Supplementary material for: Thermal tolerance in an extremophile fish from Mexico is not affected by environmental hypoxia
Source: Biol Open. 2024 Feb 5;13(2):bio060223. doi: 10.1242/bio.060223 (PMC10868586; doi:10.1242/bio.060223)
Supplement: Supplementary information [file biolopen-13-060223-s1.pdf]

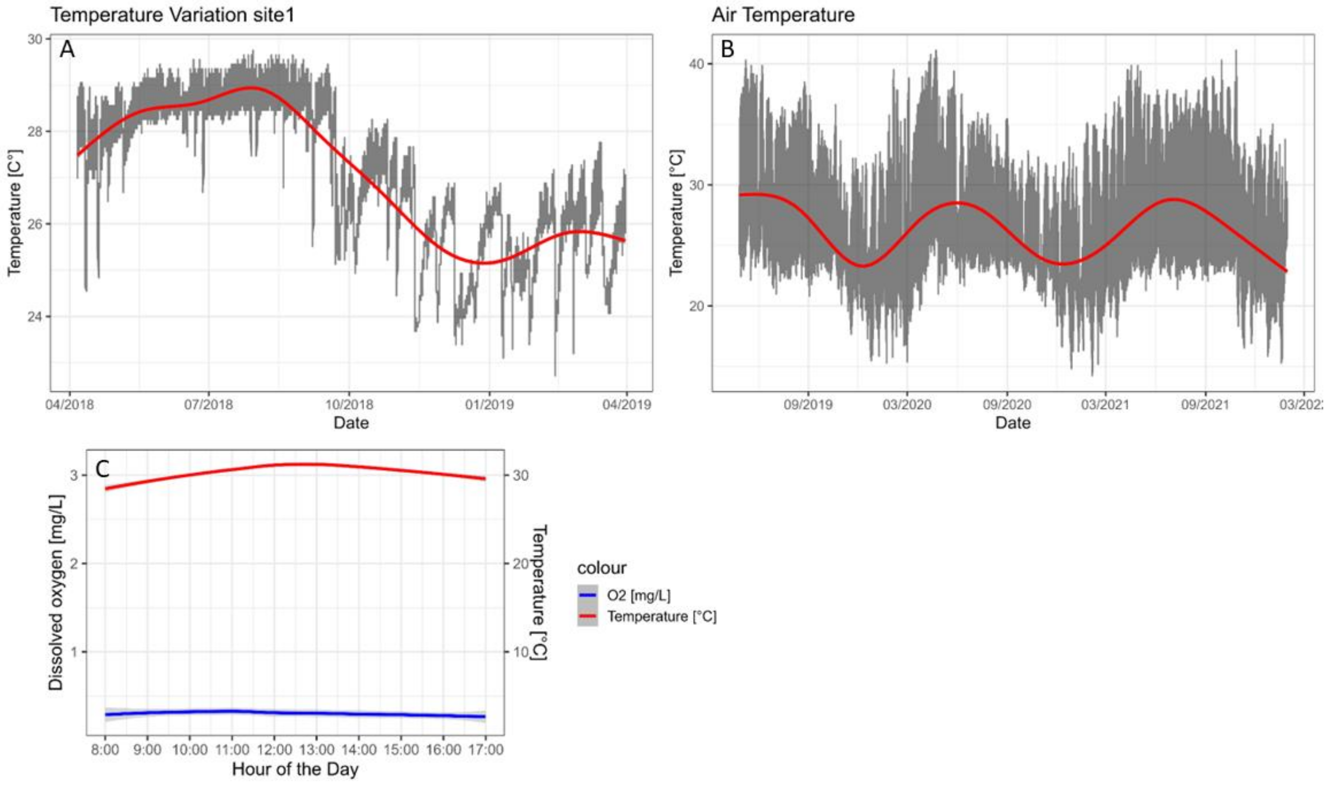

Fig. S1. Complimentary measurement data. **A:** Water temperature profile of a yearlong measurement from 04/2018 – 04/2019 at site 1. Red line indicates average temperature. Grey line indicates hourly temperature measurements. Please note the overlap in measurement period with values shown in table S1. The values in table S1 were acquired with a different temperature logger with a 10-minute measurement interval. **B**: Temperature profile of air temperature measured near field sites 1-3 from 4/2019 – 3/2022. Red line indicates average temperature. Grey line indicates individual hourly temperature measurements. **C**: Figure S3: Dissolved oxygen (blue, left axis) and temperature (red, right axis) during the day on our field sites in May 2023. Measurements were taken during the day (8:00 – 17:00) twice for every field site measured in May 2023 (site 4 & 5). Grey shaded areas indicate 95CI.

TDT Model framework

The framework supplied by (Jørgensen et al., 2021) allows for predictions based on a single measurement. As mentioned in the main text we are aware of the pitfalls that come with specific model predictions and especially the estimation of *z*. We are, however, confident that our predictions have a sufficient level of reliability for the following reasons: Firstly, our *z-values* are derived from actual experimental data (February 2023) on both species. While the range in heating rates we used to derive the values may be narrow, they fall well into the upper limit of z-values reported in the SI of Jørgensen et al. (2021, range provided: z = 1.4 – 5.8), as you would expect from species adapted to high temperatures. Secondly, we restricted our predictions to both temperature and time intervals close to the testing conditions in our experimental setting. This is crucial to avoid extrapolation uncertainties induced by predictions outside the respective thermal or temporal domain.

Table S1. TDT parameters used to predict additional CT_{max} values and maximum tolerable temperature. Note that only predictions for no oxygen treatment is shown in the manuscript as this treatment mirrors the conditions fish encounter in their habitat.

Treatment	*z*	*T_0 (C°)*	*T_C (C°)*	*dCT_{max} (C°)*	*Ramprate (C° min^{-1})*
P. sulphuraria oxygen	5.58	30	28	41.4	0.45
P. sulphuraria no oxygen	5.58	30	28	39.5	0.44
G. eurystoma oxygen	4.19	30	28	40.6	0.45
G. eurystoma no oxygen	4.19	30	28	39.0	0.44

Biology Open • Supplementary information

Table S2. Daily exposure to heat levels above certain temperatures *P. sulphuraria* and G. *eurystoma* encounter in their habitat and predictions for tolerable exposure times at those temperatures. Shown are TDT model predictions based on the results of oxygen deprived CT_{max} experiments from May 2023, as this reflects conditions fish face in their habitat. Site 2 and Site 5 were the only field sites in which temperatures above 32 °C were observed more than once in the measurement period.

**Temperature (°C)**	**Mean exposure time per day ± s.d. in habitat (minutes)**		**Predicted time of exposure tolerated (minutes)**	
	**Site 2**	**Site 5**	***G. eurystoma***	***P. sulphuraria***
32	372.0 ± 235.6	175.5 ± 106.5	199.3	267.3
32.5	279.0 ± 237.7	97.3 ± 78.9	151.4	217.5
33	234.0 ± 214.6	43.6 ± 68.9	115.0	176.9
33.5	163.5 ± 173.0	7.3 ± 24.1	87.4	143.9
34	72.0 ± 144.4	0.0 ± 0.0	66.4	117.1
34.5	43.5 ± 92.3	0.0 ± 0.0	50.4	95.3
35	19.5 ± 44.7	0.0 ± 0.0	38.3	77.5
35.5	0.0 ± 0.0	0.0 ± 0.0	29.1	63.1
36	0.0 ± 0.0	0.0 ± 0.0	22.1	51.3

References

Jørgensen, L.B., Malte, H., Ørsted, M., Klahn, N.A., Overgaard, J., 2021. A unifying model to estimate thermal tolerance limits in ectotherms across static, dynamic and fluctuating exposures to thermal stress. Sci Rep 11, 12840. https://doi.org/10.1038/s41598-021-92004-6

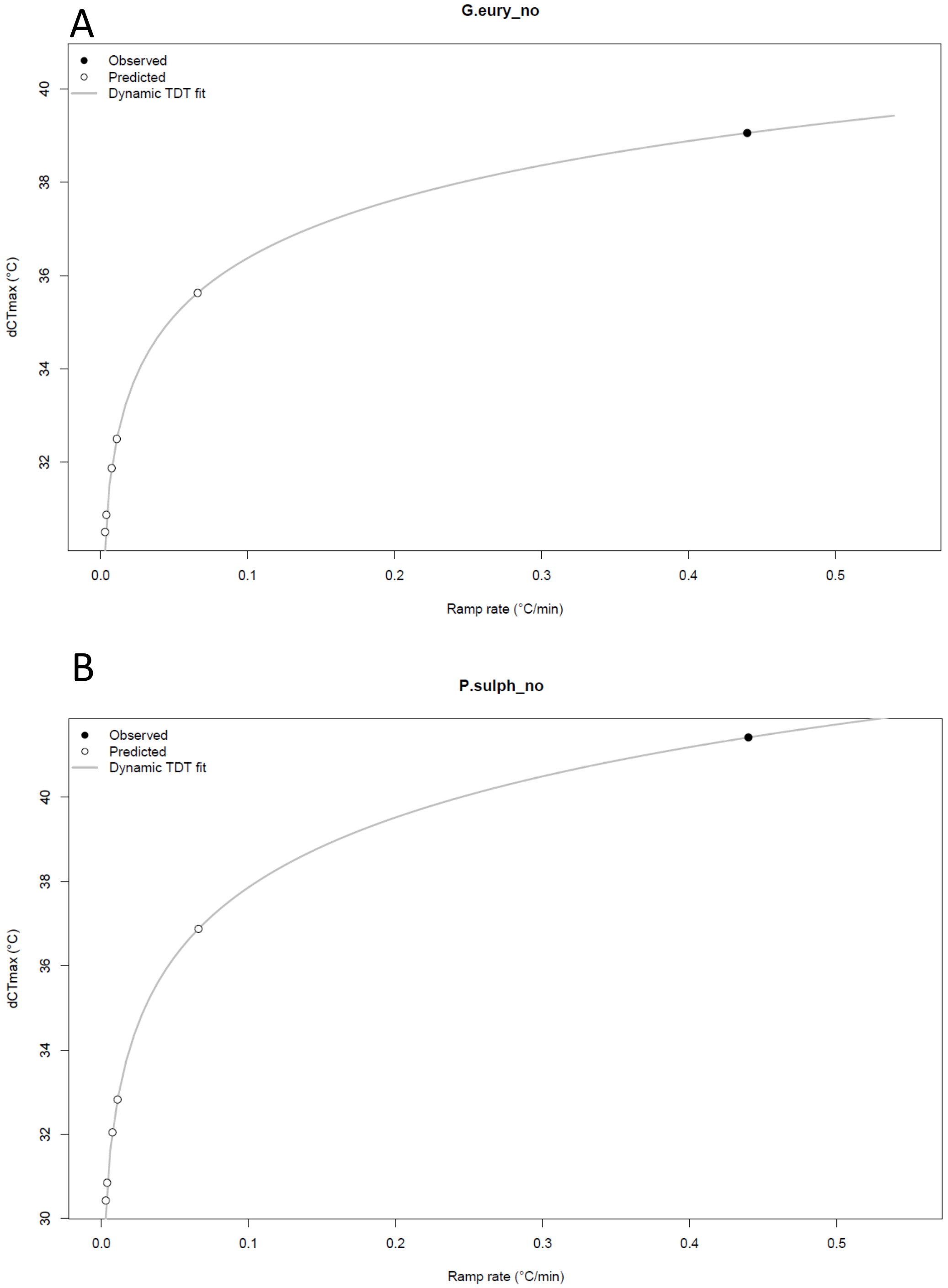

Fig. S2. TDT curves for *P. sulphuraria* (A) and *G. eurystoma* (B) under hypoxia predicted for observed heating rates in habitat (white circles) and the results of heating trials in May 2023 (black circle). For further model parameters see table S1.

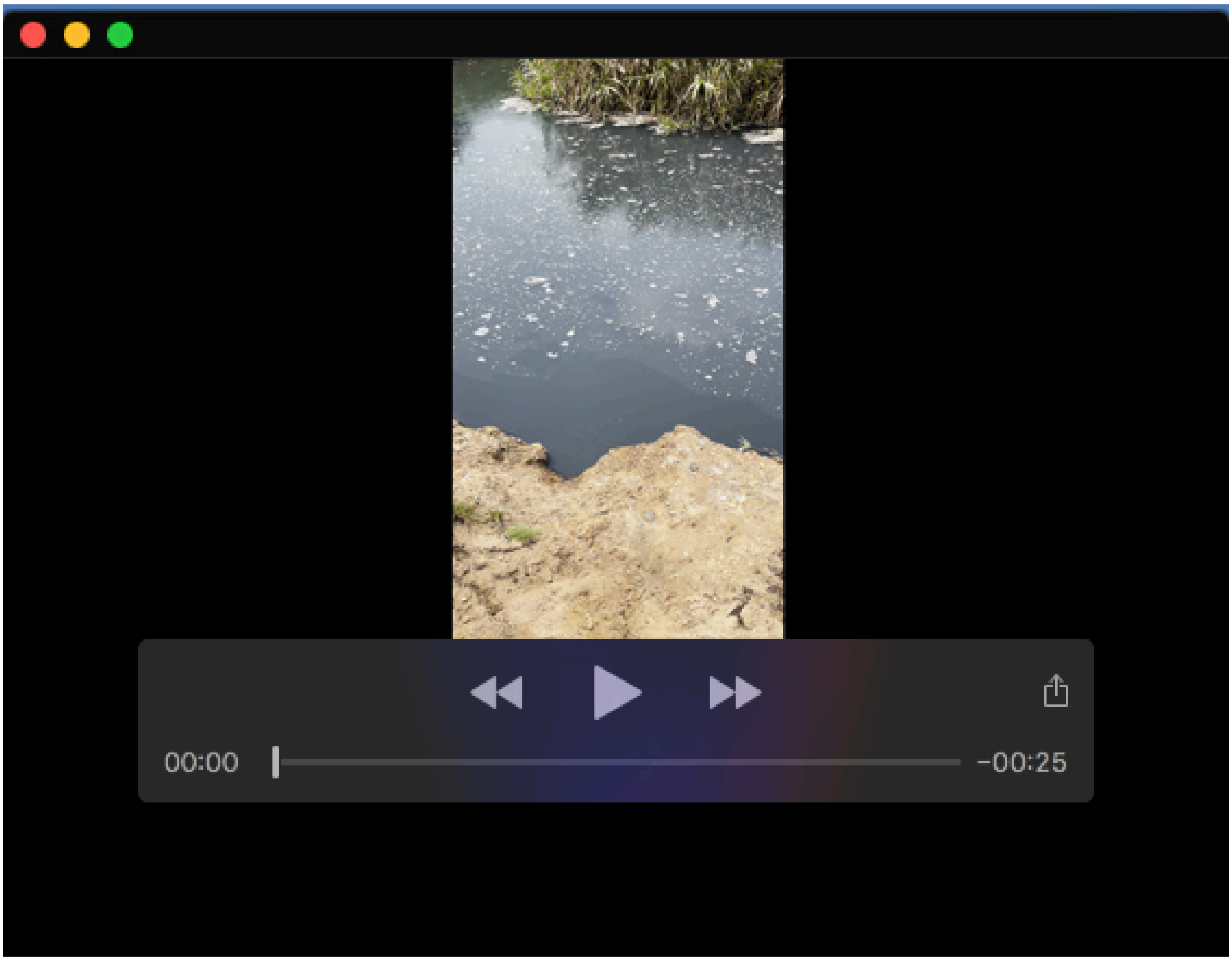

Movie 1.
